# The Role of Schools in Early Adolescents’ Mental Health: Findings From the MYRIAD Study

**DOI:** 10.1016/j.jaac.2021.02.016

**Published:** 2021-12

**Authors:** Tamsin Ford, Michelle Degli Esposti, Catherine Crane, Laura Taylor, Jesús Montero-Marín, Sarah-Jayne Blakemore, Lucy Bowes, Sarah Byford, Tim Dalgleish, Mark T. Greenberg, Elizabeth Nuthall, Alice Phillips, Anam Raja, Obioha C. Ukoumunne, Russell M. Viner, J. Mark G. Williams, Matt Allwood, Louise Aukland, Tríona Casey, Katherine De Wilde, Eleanor-Rose Farley, Nils Kappelmann, Liz Lord, Emma Medlicott, Lucy Palmer, Ariane Petit, Isobel Pryor-Nitsch, Lucy Radley, Lucy Warriner, Anna Sonley, MYRIAD Team, Saz Ahmed, Susan Ball, Marc Bennett, Nicola Dalrymple, Darren Dunning, Katie Fletcher, Lucy Foulkes, Poushali Ganguli, Cait Griffin, Kirsty Griffiths, Konstantina Komninidou, Rachel Knight, Suzannah Laws, Jovita Leung, Jenna Parker, Blanca Piera Pi-Sunyer, J. Ashok Sakhardande, Jem Shackleford, Kate Tudor, Maris Vainre, Brian Wainman, Willem Kuyken

**Affiliations:** a University of Cambridge, United Kingdom; b University College London, United Kingdom; c Cambridgeshire and Peterborough NHS Foundation Trust, Cambridgeshire, United Kingdom; d University of Oxford, United Kingdom; e King’s College London, United Kingdom; f Pennsylvania State University, State College, Pennsylvania; g University of Exeter, United Kingdom; h UCL GOS Institute of Child Health, London, United Kingdom; i University College Cork, Ireland; j Max Planck Institute of Psychiatry and International Max Planck Research School for Translational Psychiatry (IMPRS-TP), Munich, Germany; k University of Texas Southwestern Medical Center, Dallas; l University of York, United Kingdom

**Keywords:** adolescents, mental health, school climate, schools, well-being

## Abstract

**Objective:**

Recent studies suggest mental health in youths is deteriorating. The current policy in the United Kingdom emphasizes the role of schools for mental health promotion and prevention, but little data exist on what aspects of schools influence mental health in pupils. This study explored school-level influences on the mental health of young people in a large school-based sample from the United Kingdom.

**Method:**

Baseline data from a large cluster randomized controlled trial collected between 2016 and 2018 from mainstream secondary schools selected to be representative in relation to their quality rating, size, deprivation, mixed or single-sex pupil population, and country were analyzed. Participants were pupils in their first or second year of secondary school. The study assessed whether school-level factors were associated with pupil mental health.

**Results:**

The study included 26,885 pupils (response rate = 90%; age range, 11‒14 years; 55% female) attending 85 schools in the United Kingdom. Schools accounted for 2.4% (95% CI: 2.0%‒2.8%; *p* < .0001) of the variation in psychopathology, 1.6% (95% CI: 1.2%‒2.1%; *p* < .0001) of depression, and 1.4% (95% CI: 1.0%‒1.7%; *p* < .0001) of well-being. Schools in urban locations, with a higher percentage of free school meals and of White British, were associated with poorer pupil mental health. A more positive school climate was associated with better mental health.

**Conclusion:**

School-level variables, primarily related to contextual factors, characteristics of pupil population, and school climate, explain a small but significant amount of variability in mental health of young people. This information might be used to identify schools that are in need of more resources to support mental health of young people.

**Clinical trial registration information:**

MYRIAD: My Resilience in Adolescence, a Study Examining the Effectiveness and Cost-Effectiveness of a Mindfulness Training Programme in Schools Compared With Normal School Provision; https://www.isrctn.com/; 86619085.

A significant proportion of children and adolescents are affected by mental health conditions, with some studies suggesting increased anxiety, depression, and self-injury in young people.^1^^,^^2^ Approximately 75% of adults who experience poor mental health in adulthood first experience difficulties before age 18.^3^ People affected by mental health problems during this developmental window pay a heavy price in terms of poorer educational and occupational outcomes, relationship difficulties, and recurring depression.^4^^,^^5^ So it is particularly worrying that evidence suggests worse outcomes in recent cohorts, even before the 2019 novel coronavirus disease (COVID-19) pandemic.^6^

Different aspects of school experience may influence mental health and well-being in young people through various mechanisms (see Figure S1, available online). Some factors, such as the experience of pervasive bullying in the school environment, may directly impact a young person’s mental health, while others may act indirectly—for instance, the quality and character of the school as an institution, often referred to as school climate.^7^ Furthermore, some potential influences will be outside the school’s control, for example, the socioeconomic profile of the school catchment area, yet may still be important influences on pupils’ mental health and therefore could be an indicator of need for additional resources.^8^ Given the long-term and near-universal access that education provides, schools are a potentially powerful setting for delivering effective interventions to support well-being, to prevent mental health problems, and to triage identified difficulties.^9^ Mental health provision in schools is highly variable within as well as between countries and is a current policy focus in the United Kingdom, which traditionally has not had a strong school-based mental health service.^10^

The limited literature suggests that school has a small but significant influence on pupils’ mental health, explaining 1%–6% of the variation.^2^^,^^11^ For example, the sense of school-connectedness is associated with mental health and educational outcomes.^7^ A relationship between school-level sense of community and the well-being of the pupils has been observed^11^: young adolescents attending schools with higher levels of bullying are more likely to have poor mental health,^12^ while school-level collective efficacy is more strongly related to adolescent alcohol use than neighbourhood-level collective efficacy.^13^

Nevertheless, schools operate in a wider structural or socioeconomic context, with factors such as deprivation directly and consistently affecting mental health.^14^ Even though schools may not be able to alter the broader context of the catchment area from which their pupils come, there is some evidence that they can still affect mental health of pupils over and above these powerful structural influences. For example, the US National Longitudinal Study of Adolescent Health suggested that school-level variables influence symptoms of depression in adolescents over and above structural neighborhood factors.^15^ Similarly, a Scottish cohort study that followed subjects from childhood into middle age reported school-level effects on adult self-rated health, after accounting for structural socioeconomic factors.^16^ Together, this limited literature suggests that while schools operate in a wider context, they may nonetheless have a specific role to play in the mental health of their students. At minimum, understanding these factors and mechanisms could help target prevention and intervention, using the school as a vehicle for evidence-based programs.^8^

In this study, we aimed to determine the extent to which variability in mental health of pupils is attributable to schools and describe which school-related factors are associated with pupils’ mental health, including wider structural socioeconomic factors (urbanity, area-level deprivation), characteristics of the school community (free school meals, special educational needs or disabilities support, ethnicity), and operational features of the school (school size, pupil-to-teacher ratio, mixed/single sex, school quality, social and emotional learning [SEL] provision, and school climate). We used a large (N = 26,885) sample of pupils attending 85 secondary schools from the United Kingdom, collecting data on psychopathology, depression, and well-being using well-established continuous measures.

## Method

This study is a cross-sectional secondary analysis of baseline data collected as part of the MYRIAD Project, a cluster randomized controlled trial evaluating whether school-based mindfulness training improves mental health of young people (ISRCTN Registry reference 86619085).^17^ Data used in this study were collected before randomization of the schools and at least 1 year before the delivery of any intervention, and thus the current analysis is not part of the intervention study. The rationale for the trial is explained in the study protocol.^17^ Administrative data were linked and collected from the 85 UK schools participating in the trial (75 in England, 4 in Northern Ireland, 3 in Scotland, and 3 in Wales), 739 teachers, and 26,885 pupils 11‒14 years of age who were in their first or second year of secondary school during the 2016–2017 and 2017–2018 academic years. The study was approved by the University of Oxford Medical Sciences Division Ethics Committee.

We recruited schools (N = 85) in 2 cohorts: pupils provided baseline data in the academic year 2016–2017 (cohort 1; n = 13) or 2017–2018 (cohort 2; n = 72). Participant flow is described in Figure S2, available online, and additional details about study design, recruitment, and procedure are provided in Supplement 1, available online. All mainstream UK secondary schools, including private schools, were eligible if they had a substantive appointed headteacher, had not been judged inadequate in their most recent official inspection (to mitigate any risk for trial implementation), and had a strategy and structure in place for delivery of SEL (which is usually taught in Personal, Social, Health, and Economic Education in England; see Supplement 2, available online).

Three groups of school-level factors were identified: factors that related to the broader school context; characteristics of the school community, and operational features of the school (Figure S1, available online). Measures that were directly comparable across England, Northern Ireland, Scotland, and Wales were selected, where possible; otherwise, measures were mapped to their English equivalent. Pupil-level measures included mental health and demographics.

The broader school context represented wider structural socioeconomic factors in the area which the school was located, including whether a school was in a rural or urban area, and area-level deprivation (Index of Multiple Deprivation decile rating; see Supplements 1 and 2, available online) obtained by linking to the school’s post code. In terms of characteristics of school community, we obtained the number of pupils in each school who were eligible for free school meals (as an indicator of socioeconomic status), received support for special educational needs or disabilities, and were White British (see Supplement 2, available online). The operational features of the school were the total number of pupils and the pupil-to-teacher ratio for all schools, which were also classified as mixed or single sex. An ordinal variable described overall school quality based on inspection ratings (Office for Standards in Education for England; see Supplement 2, available online), which was analyzed as an ordinal categorical variable (0 = requires improvement; 1 = good; 2 = outstanding). SEL provision was assessed against 16 quality indicators via a semistructured interview with the staff member with overall responsibility for the subject (see Supplement 2, available online). Participating teachers within each school completed 3 subscales from the Alaska School Climate and Connectedness Survey (School Leadership and Involvement, Staff Attitudes, and Respectful Climate) to provide a rating of school climate (data sources and further details are provided in Supplements 1 and 2, available online).

Mental health of pupils (eg, psychopathology, depression, and well-being) was measured with 3 validated self-report questionnaires: the Strengths and Difficulties Questionnaire (SDQ),^18^ the Center for Epidemiologic Studies-Depression (CES-D) Scale,^19^ and the Warwick-Edinburgh Mental Well-Being Scale (WEMWBS).^20^ The SDQ is a 25-item questionnaire that assesses psychopathology over the previous 6 months and is validated for use in school-age children. The 5 subscales assess emotional symptoms, conduct problems, hyperactivity/inattention, peer problems, and prosocial behavior. We report a total score (range, 0–40) derived by summing the first 4 subscales, where higher scores indicate higher levels of psychopathology. The CES-D Scale is a 20-item questionnaire that assesses depressive symptoms and has been validated for use in adolescents. Each item is rated on a scale from 0 to 3, yielding a total score between 0 and 60, where higher scores indicate more symptoms of depression. The WEMWBS is a 14-item measure assessing mental well-being that has been validated for use in adolescents. Each item is scored on a scale from 1 to 5, yielding a total score between 14 and 70 (higher scores indicate greater well-being). Pupils also provided data on their gender (male, female, other/prefer not to say) and ethnicity (White, Asian, Black, and Mixed and other ethnic minorities [eg, Arab]). Pupils’ ages were obtained from school.

### Analytic Approach

Multilevel linear regression models were fitted using lme4 in R 3.5.2^21^ to estimate school-level variance in pupils’ mental health—psychopathology, depression, and well-being, which were analyzed separately throughout. We reported the intracluster (intraschool) correlation coefficient (ICC), which is the proportion of the total variance in the outcome attributed at the school level. We fitted variance components (empty) multilevel models with no fixed predictors to estimate the ICCs for pupils’ mental health. We then fitted multilevel models to estimate the ICCs for pupils’ mental health, while using pupils’ gender, age, and ethnicity as predictors to control differences across clusters on these individual level variables. The 95% CIs and *p* values for the ICCs were obtained using nonparametric bootstrapping.

We explored whether school factors accounted for any school-level variation in pupils’ mental health. First, we examined the unique associations between each school factor and pupils’ mental health, while accounting for pupils’ nesting within schools using multilevel regression models, with random intercepts only. Next, we fitted our 3 main multilevel models corresponding to the 3 types of school-level factors, as described above and in Figure S1, available online. School-related factors that belonged to the same type were entered as covariates in the same multivariable model. We further adjusted for gender, age, and ethnicity at the pupil level to verify that the associations between school factors and pupils’ mental health remained stable. We report sensitivity analyses to test for possible differences between pupils who were in their first year of secondary school compared with pupils who were in their second year as well as between pupils scoring above and below cutoff for probable caseness of psychopathology. Thus, we stratified by year group and separately by SDQ caseness (SDQ ≥18),^22^ and we reran the analyses on the different subsamples and descriptively compared them to spot any potential substantial difference. We also used a similar approach to run restricted subanalyses for schools in England only (n = 75 schools; n = 24,842 pupils).

To assist the interpretation of results, we grand mean centered all continuous pupil (age) and school factors. Multilevel models were fitted using restricted maximum likelihood estimation, and model assumptions and fit were checked via absolute model fit indices (root mean square error of approximation <0.10 and standardized root mean square residual <0.08).^23^ We conducted complete case analyses, as there were minimal missing data (range, 0.0%‒2.8%) (Table S1 and Table S2, available online), and used 2-sided contrasts with a significance level of .05. Although the study was exploratory, we checked for inflation of type I errors from multiple testing by controlling for the false discovery rate and calculating Benjamini-Hochberg adjusted *p* values.^24^

## Results

Table 1 presents the characteristics of the sample of schools and pupils. Most schools were in an urban area (85%). Inspection quality ratings suggested that 17% required improvement, 58% were good, and 25% were outstanding. There was, however, considerable variation between schools in terms of pupil ethnicity, levels of pupil eligibility for free school meals, and receipt of support for special educational needs or disabilities. School area-level deprivation also differed markedly between schools, and there was variation between schools in size, pupil-to-teacher ratio, and SEL provision. Eleven (13%) schools were single gender, all of which were girls’ schools. Mental health of pupils was in line with national estimates for this age group (range, 10–14 years old).^19^^,^^20^^,^^22^

**Table 1:** Characteristics of Schools (N = 85) and Pupils (N = 26,885)

Characteristic	Value	
School context		
Urbanicity, n (%)		
Rural	13	(15.29)
Urban	72	(84.71)
Area-level deprivation, IMD, mean (SD)	5.82	(2.73)
Characteristics of school community		
Percentage of pupils eligible for free school meals, mean (SD)	12.21	(9.33)
Percentage of pupils receiving SEND support, mean (SD)	9.99	(5.56)
Percentage of pupils who are White British, mean (SD)	76.15	(24.58)
Operational features of the school		
Mixed or single sex school (n, %)		
Mixed	74	(87.06)
Female only	11	(12.94)
Number of pupils, mean (SD)	1016.15	(337.02)
Pupil-to-teacher ratio, mean (SD)	15.92	(1.85)
School quality, OFSTED rating^a^, n (%)		
Requires improvement	14	(17.28)
Good	47	(58.02)
Outstanding	20	(24.69)
SEL provision quality rating, mean (SD)	11.99	(2.58)
Teacher-rated school climate, SCCS, mean (SD)	3.94	(0.28)
Pupil sociodemographics		
Gender, n (%)		
Female	14,499	(55.25)
Male	11,201	(42.68)
Other/prefer not to say	543	(2.07)
Age, y, mean (range)	12.20	(10.90–14.73)
Ethnicity, n (%)		
White British	19,652	(75.18)
Asian	2,731	(10.45)
Black	1,432	(5.48)
Mixed and other ethnic minorities (eg, Arab)	2,325	(8.89)
Pupil mental health		
Psychopathology, SDQ^b^, mean (SD)	11.85	(6.50)
Normal, n (%)	17,781	(67.60)
Borderline, n (%)	3,309	(12.58)
High, n (%)	1,657	(6.30)
Very high, n (%)	3,554	(13.51)
Depression, CES-D^c^, mean (SD)	13.62	(10.06)
Normal, n (%)	17,844	(67.21)
At risk, n (%)	5,910	(22.26)
Caseness, n (%)	2,796	(10.53)
Well-being, WEMWBS, mean (SD)	49.57	(9.87)

Note: Sample size (n) and percentage (%) are given for categorical variables, and mean and SD are given for continuous variables; complete sample (N = 85 schools; N = 26,885 pupils), but number varies owing to missing data. CES-D = Center for Epidemiological Studies-Depression; IMD = index of multiple deprivation; OFSTED = Office for Standards in Education; SCCS = School Climate and Connectedness Survey; SDQ = Strengths and Difficulties Questionnaire; SEL = social and emotional learning; SEND = special educational needs and disability; WEMWBS = Warwick-Edinburgh Mental Well-Being Scale.

a OFSTED operates in England only.

b SDQ cutoff points: normal (0–14); borderline (15–17); high (18–19); very high (20–40).^25^

c CES-D cutoff points: low (0–15); at risk of depression (16–27); caseness (28–60).^23^

A small but statistically significant proportion of the total variance in pupils’ mental health was explained at the school level (Table 2). The amount of variance attributable to schools was highest for psychopathology at 2.4% (95% CI: 2.0%–2.8%), followed by depression at 1.6% (95% CI: 1.2%–2.1%) and well-being at 1.4% (95% CI: 1.0%–1.7%). All 3 ICCs were similar after including pupils’ individual characteristics (gender, age, and ethnicity) (Table 2) as predictors in the model. A sensitivity analysis showed no difference between pupils who were in their first year of secondary school compared with pupils who were in their second year or between pupils’ scoring above and below cutoff for caseness of psychopathology (Table S3 and Table S4, available online). Restricted analyses for England showed a similar pattern of results (Table S5, available online).

**Table 2:** Intraclass Correlation Coefficients for School-Level Variance of Pupils’ Mental Health

Pupil’s mental health	n		Unadjusted models			n		Adjusted models for pupil’s age, gender, and ethnicity		
	Pupils	Schools	ICC	(95% CI)	*p*	Pupils	Schools	ICC	(95% CI)	*p*
Psychopathology, SDQ	26,303	85	0.024	(0.020 to 0.028)	< .0001	26,127	85	0.022	(0.017 to 0.026)	< .0001
Depression, CES-D Scale	26,549	85	0.016	(0.012 to 0.021)	< .0001	26,078	85	0.015	(0.011 to 0.018)	< .0001
Well-being, WEMWBS	26,463	85	0.014	(0.010 to 0.017)	< .0001	26,073	85	0.014	(0.010 to 0.017)	< .0001

Note: Multilevel models are based on complete case analysis; total sample (N = 85 schools; N = 26,885 pupils), but number varies owing to missing data. CES-D = Center for Epidemiologic Studies-Depression; ICC = intraclass correlation coefficient; SDQ = Strengths and Difficulties Questionnaire; WEMWBS = Warwick-Edinburgh Mental Well-Being Scale.

Associations for the 3 types of school-related factors and psychopathology, depression, and psychological well-being in pupils are presented in Table 3 (the unique associations are presented in Table 4). Among school context variables, urban location was positively associated with depression in pupils (regression coefficient [B] = 0.90; 95% CI: 0.05 to 1.74; *p* = .04), even when adjusting for school area-level deprivation and individual confounders. School area-level deprivation, in contrast, was not associated with psychopathology, depression, or psychological well-being in pupils, suggesting better mental health and well-being among pupils attending schools located in rural areas, regardless of whether the area surrounding the school is affluent or deprived.

**Table 3:** Results From Multilevel Models With Random Intercepts Showing Grouped Associations Between Different Types of School Factors and Pupils’ Mental Health

School factors	Psychopathology (SDQ)						Depression (CES-D Scale)						Well-being (WEMWBS)					
	Unadjusted models			Adjusted models for pupil’s age, gender, and ethnicity			Unadjusted models			Adjusted models for pupil’s age, gender, and ethnicity			Unadjusted models			Adjusted models for pupil’s age, gender, and ethnicity		
	Coefficient	(95% CI)	*p*	Coefficient	(95% CI)	*p*	Coefficient	(95% CI)	*p*	Coefficient	(95% CI)	*p*	Coefficient	(95% CI)	*p*	Coefficient	(95% CI)	*p*
**Broader school context**																		
Urban vs rural	0.36	(−0.29 to 1.01)	.29	0.49	(−0.12 to 1.10)	.12	0.90	(0.05 to 1.74)	.040	0.89	(0.09 to 1.69)	.032	−0.65	(−1.44 to 0.14)	.11	−0.73	(−1.51 to 0.05)	.07
Area-level deprivation	−0.07	(−0.15 to 0.02)	.13	−0.08	(−0.16 to 0.00)	.055	−0.06	(−0.17 to 0.05)	.30	−0.06	(−0.17 to 0.04)	.26	−0.01	(−0.11 to 0.10)	.87	0.00	(−0.10 to 0.10)	.99
**Characteristics of school community**																		
Pupils eligible for free school meals (%)	0.06	(0.03 to 0.09)	< .001	0.06	(0.03 to 0.09)	< .001	0.04	(0.00 to 0.09)	.05	0.05	(0.01 to 0.09)	.011	−0.03	(−0.06 to 0.01)	.17	−0.04	(−0.07 to 0.00)	.041
SEND support (%)	−0.01	(−0.06 to 0.04)	.70	0.00	(−0.05 to 0.04)	.89	−0.04	(−0.11 to 0.03)	.26	−0.03	(−0.09 to 0.03)	.36	0.01	(−0.05 to 0.07)	.63	0.01	(−0.05 to 0.06)	.86
Ethnicity of pupils (%): White	0.02	(0.01 to 0.03)	< .001	0.01	(0.00 to 0.02)	.054	0.01	(−0.01 to 0.02)	.33	0.01	(0.00 to 0.02)	.10	−0.02	(−0.03 to −0.01)	.001	−0.02	(−0.03 to −0.01)	.005
**Operational features of the school**																		
Mixed or single-sex school	−0.01	(−0.77 to 0.75)	.98	0.00	−0.73 to 0.73)	.99	0.80	(−0.22 to 1.82)	.13	−0.16	(−1.16 to 0.84)	.76	0.01	(−0.95 to 0.97)	.99	0.69	(−0.25 to 1.63)	.15
School quality	−0.13	(−0.66 to 0.40)	.62	−0.04	(−0.53 to 0.45)	.87	0.02	(−0.69 to 0.72)	.97	0.09	(−0.60 to 0.77)	.80	0.40	(−0.27 to 1.06)	.24	0.27	(−0.35 to 0.90)	.40
School size (per 100 pupils)	−0.06	(−0.14 to 0.02)	.15	−0.06	(−0.13 to 0.02)	.16	−0.11	(−0.22 to 0.01)	.07	−0.10	(−0.22 to 0.02)	.08	0.03	(−0.06 to 0.13)	.53	0.03	(−0.07 to 0.13)	.60
Pupil-to-teacher ratio	−0.06	(−0.19 to 0.08)	.44	−0.06	(−0.19 to 0.08)	.40	−0.05	(−0.25 to 0.14)	.58	−0.08	(−0.28 to 0.12)	.40	0.00	(−0.18 to 0.17)	.98	0.04	(−0.14 to 0.21)	.69
SEL provision	0.00	(−0.10 to 0.09)	.92	−0.01	(−0.09 to 0.07)	.83	−0.02	(−0.14 to 0.09)	.71	−0.02	(−0.13 to 0.10)	.81	−0.05	(−0.17 to 0.07)	.41	−0.04	(−0.16 to 0.08)	.49
Teacher-rated SCCS	−1.11	(−2.19 to −0.03)	.046	−1.22	(−2.22 to −0.22)	.020	−1.19	(−2.64 to 0.26)	.11	−1.20	(−2.61 to 0.21)	.10	0.58	(−0.77 to 1.94)	.40	0.69	(−0.60 to 1.99)	.30

Note: Estimates are based on complete case analyses; total sample (N = 85 schools; N = 26,885 pupils), but N varies owing to missing data. CES-D = Center for Epidemiologic Studies-Depression; SCCS = School Climate and Connectedness Survey; SDQ = Strengths and Difficulties Questionnaire; SEL = social and emotional learning; SEND = special educational needs and disability; WEMWBS = Warwick-Edinburgh Mental Well-Being Scale.

**Table 4:** Unique Associations From Multilevel Models With Random Intercepts Between School Factors and Pupil’s Mental Health

School factors	Psychopathology (SDQ)						Depression (CES-D Scale)						Well-being (WEMWBS)					
	Unadjusted models			Adjusted models for pupil’s age, gender, and ethnicity			Unadjusted models			Adjusted models for pupil’s age, gender, and ethnicity			Unadjusted models			Adjusted models for pupil’s age, gender, and ethnicity		
	Coefficient	(95% CI)	*p*	Coefficient	(95% CI)	*p*	Coefficient	(95% CI)	*p*	Coefficient	(95% CI)	*p*	Coefficient	(95% CI)	*p*	Coefficient	(95% CI)	*p*
Urban vs rural	0.49	(−0.14 to 1.12)	.13	0.64	(0.05 to 1.24)	.037	1.02	(0.20 to 1.83)	.017	1.01	(0.24 to 1.79)	.012	−0.63	(−1.39 to 0.13)	.11	−0.73	(−1.48 to 0.02)	.06
Area-level deprivation	−0.08	(−0.16 to 0.00)	.06	−0.10	(−0.18 to −0.02)	.018	−0.09	(−0.20 to 0.02)	.11	−0.09	(−0.20 to 0.01)	.09	0.01	(−0.09 to 0.12)	.79	0.03	(−0.08 to 0.13)	.62
Pupils eligible for free school meals (%)	0.03	(0.01 to 0.06)	.016	0.03	(0.01 to 0.06)	.010	0.02	(−0.02 to 0.05)	.29	0.02	(−0.01 to 0.05)	.26	0.00	(−0.03 to 0.03)	.89	−0.01	(−0.04 to 0.02)	.65
SEND support (%)	0.02	(−0.02 to 0.07)	.32	0.02	(−0.02 to 0.07)	.28	−0.01	(−0.07 to 0.05)	.75	0.00	(−0.06 to 0.06)	.94	−0.01	(−0.06 to 0.05)	.83	−0.01	(−0.07 to 0.04)	.65
Ethnicity of pupils (%): White	0.01	(0.00 to 0.02)	.048	0.00	(−0.01 to 0.01)	.63	0.00	(−0.01 to 0.01)	.77	0.01	(−0.01 to 0.02)	.42	−0.02	(−0.03 to −0.01)	.004	−0.01	(−0.02 to 0.00)	.032
Mixed or single sex school	−0.31	(−0.99 to 0.37)	.37	−0.19	(−0.85 to 0.47)	.57	0.61	(−0.28 to 1.50)	.18	−0.24	(−1.11 to 0.63)	.59	0.38	(−0.44 to 1.20)	.37	0.95	(0.14 to 1.75)	.024
School quality	−0.48	(−0.83 to −0.13)	.009	−0.41	(−0.75 to −0.07)	.019	−0.32	(−0.80 to 0.16)	.20	−0.45	(−0.90 to −0.01)	.06	0.55	(0.12 to 0.97)	.014	0.61	(0.19 to 1.02)	.005
School size (per 100 pupils)	−0.06	(−0.13 to 0.00)	.071	−0.06	(−0.13 to 0.00)	.054	−0.10	(−0.19 to −0.01)	.035	−0.09	(−0.18 to −0.01)	.032	0.03	(−0.05 to 0.12)	.43	0.03	(−0.05 to 0.11)	.51
Pupil-to-teacher ratio	−0.1	(−0.23 to 0.03)	.14	−0.09	(−0.22 to 0.03)	.16	−0.09	(−0.27 to 0.08)	.31	−0.09	(−0.26 to 0.08)	.29	0.04	(−0.12 to 0.20)	.62	0.06	(−0.10 to 0.22)	.46
SEL provision	−0.02	(−0.11 to 0.07)	.67	−0.02	(−0.10 to 0.07)	.72	−0.01	(−0.13 to 0.11)	.84	−0.02	(−0.13 to 0.10)	.75	−0.04	(−0.15 to 0.07)	.49	−0.03	(−0.13 to 0.08)	.65
Teacher-rated SCCS	−1.48	(−2.27 to −0.70)	< .001	−1.35	(−2.10 to −0.59)	< .001	−1.22	(−2.30 to −0.13)	.030	−1.45	(−2.47 to −0.44)	.006	1.31	(0.32 to 2.29)	.011	1.50	(0.54 to 2.47)	.003

Note: Estimates are based on complete case analyses; total sample (N = 85 schools; N = 26,885 pupils), but N varies owing to missing data. CES-D = Center for Epidemiologic Studies-Depression; SCCS = School Climate and Connectedness Survey; SEL = social and emotional learning; SEND = special educational needs and disability; SDQ = Strengths and Difficulties Questionnaire; WEMWBS = Warwick-Edinburgh Mental Well-Being Scale.

In the school community, a higher percentage of free school meal eligibility was associated with higher levels of psychopathology in pupils (B = 0.06; 95% CI: 0.03 to 0.09; *p* < .001), even while accounting for the percentage of pupils receiving special educational needs or disabilities support and school ethnic composition. A higher proportion of White British pupils in schools was correlated with higher levels of psychopathology (B = 0.02; 95% CI: 0.01 to 0.03; *p* < .001) and lower levels of well-being (B = −0.02; 95% CI: −0.03 to −0.01; *p* = .001), when accounting for the percentage of pupils receiving special educational needs or disabilities support and free school meal eligibility. The association with well-being remained after adjusting for individual-level confounders but was attenuated for psychopathology (B = 0.01; 95% CI: 0.00 to 0.032; *p* = .054). There was no association between the percentage of pupils receiving support for special educational needs or disabilities and pupils’ mental health.

Among operational features of the school, teacher-rated school climate was the only school-level factor to show associations with mental health of pupils. In schools with a more positive school climate, pupils reported less psychopathology, less depression, and greater mental well-being (Table 4). Teacher-rated positive school climate remained associated with lower levels of psychopathology (B = −1.11; 95% CI: −2.19 to −0.03; *p* = .046) after adjusting for other operational variables (mixed/single sex school, school quality, school size, pupil-to-teacher ratio, and SEL provision) and after adjusting for individual confounders (Tables 2 and 3). However, the associations between school climate and depression or well-being were attenuated when adjusted for other operational variables and confounders (Tables 2 and 3). Some associations were attenuated when using *p* values adjusted for multiple testing (eg, school urbanity and higher depression), but differences were minimal (Table 5). Results also did not significantly change when restricting the analyses to England only (see Table S6, available online). The only potentially meaningful difference was that school size was negatively associated with higher levels of depression in English schools, after controlling for individual characteristics.

**Table 5:** Results From Multilevel Models With Random Intercepts Showing Grouped Associations Between Different Types of School Factors and Pupils’ Mental Health Using Adjusted *p* Values for Multiple Comparisons

School factors	Psychopathology (SDQ)						Depression (CES-D Scale)						Well-being (WEMWBS)					
	Unadjusted models			Adjusted models for pupil’s age, gender, and ethnicity			Unadjusted models			Adjusted models for pupil’s age, gender, and ethnicity			Unadjusted models			Adjusted models for pupil’s age, gender, and ethnicity		
	Coefficient	(95% CI)	B-H *p*	Coefficient	(95% CI)	B-H *p*	Coefficient	(95% CI)	B-H *p*	Coefficient	(95% CI)	B-H *p*	Coefficient	(95% CI)	B-H *p*	Coefficient	(95% CI)	B-H *p*
**Broader school context**																		
Urban vs rural	0.36	(−0.29 to 1.01)	.40	0.49	(−0.12 to 1.10)	.20	0.90	(0.05 to 1.74)	.080	0.89	(0.09 to 1.69)	.065	−0.65	(−1.44 to 0.14)	.19	−0.73	(−1.51 to 0.05)	.13
Area-level deprivation	−0.07	(−0.15 to 0.02)	.21	−0.08	(−0.16 to 0.00)	.10	−0.06	(−0.17 to 0.05)	.41	−0.06	(−0.17 to 0.04)	.37	−0.01	(−0.11 to 0.10)	.94	0.00	(−0.10 to 0.10)	.99
**Characteristics of school community**																		
Pupils eligible for free school meals (%)	0.06	(0.03 to 0.09)	< .001	0.06	(0.03 to 0.09)	< .001	0.04	(0.00 to 0.09)	.10	0.05	(0.01 to 0.09)	.023	−0.03	(−0.06 to 0.01)	.27	−0.04	(−0.07 to 0.00)	.081
SEND support (%)	−0.01	(−0.06 to 0.04)	.81	0.00	(−0.05 to 0.04)	.96	−0.04	(−0.11 to 0.03)	.37	−0.03	(−0.09 to 0.03)	.48	0.01	(−0.05 to 0.07)	.75	0.01	(−0.05 to 0.06)	.94
Ethnicity of pupils (%): White	0.02	(0.01 to 0.03)	.002	0.01	(0.00 to 0.02)	.10	0.01	(−0.01 to 0.02)	.45	0.01	(0.00 to 0.02)	.18	−0.02	(−0.03 to −0.01)	.002	−0.02	(−0.03 to −0.01)	.010
**Operational features of the school**																		
Mixed or single-sex school	−0.01	(−0.77 to 0.75)	.99	0.00	(−0.72 to 0.71)	.99	0.80	(−0.23 to 1.82)	.22	−0.16	(−1.17 to 0.85)	.86	0.01	(−0.95 to 0.97)	.99	0.69	(−0.24 to 1.62)	.25
School quality	−0.13	(−0.65 to 0.39)	.74	−0.04	(−0.53 to 0.45)	.94	0.02	(−0.69 to 0.72)	.99	0.09	(−0.60 to 0.77)	.90	0.40	(−0.26 to 1.06)	.36	0.27	(−0.36 to 0.91)	.51
School size (per 100 pupils)	−0.06	(−0.14 to 0.02)	.25	−0.06	(−0.13 to 0.02)	.26	−0.11	(−0.22 to 0.01)	.12	−0.10	(−0.21 to 0.01)	.14	0.03	(−0.07 to 0.14)	.64	0.03	(−0.07 to 0.13)	.72
Pupil-to-teacher ratio	−0.06	(−0.20 to 0.09)	.55	−0.06	(−0.19 to 0.08)	.51	−0.05	(−0.25 to 0.14)	.71	−0.08	(−0.27 to 0.11)	.51	0.00	(−0.18 to 0.18)	.99	0.04	(−0.14 to 0.21)	.81
SEL provision	−0.01	(−0.10 to 0.09)	.97	−0.01	(−0.10 to 0.08)	.92	−0.02	(−0.15 to 0.10)	.82	−0.02	(−0.14 to 0.11)	.90	−0.05	(−0.17 to 0.07)	.51	−0.04	(−0.15 to 0.07)	.60
Teacher-rated SCCS	−1.11	(−2.18 to −0.04)	.09	−1.22	(−2.22 to −0.22)	.041	−1.19	(−2.64 to 0.26)	.19	−1.20	(−2.6 to 0.21)	.18	0.58	(−0.77 to 1.94)	.51	0.69	(−0.61 to 1.99)	.41

Note: Estimates are based on complete case analyses; total sample (N = 85 schools; N = 26,885 pupils), but N varies owing to missing data. B-H adjusted p values are presented to control for false discovery rate from multiple testing. B-H = Benjamini-Hochberg; CES-D = Center for Epidemiologic Studies-Depression; SCCS = School Climate and Connectedness Survey; SEL = social and emotional learning; SEND = special educational needs and disability; SDQ = Strengths and Difficulties Questionnaire; WEMWBS = Warwick-Edinburgh Mental Well-Being Scale.

To assess whether these relationships were influenced by how long pupils had been in the school, we compared pupil year groups (eg, pupils in their first year who had recently joined the school and pupils in their second year who had typically been immersed in the school culture for 12 months longer). We found no evidence to suggest that there were systematic differences in school-level variance across these 2-year groups.

## Discussion

Given the increasing recent focus of policy makers and researchers on the role of schools in mental health of young people,^9^^,^^10^ we examined the extent to which variation in mental health of young people could be explained by variables operating at the school level in current secondary schools in the United Kingdom. We considered wider structural socioeconomic factors, characteristics of the school community, and operational features of the school. We used data obtained from a sample of 26,885 pupils attending 85 schools from across the United Kingdom.

Consistent with the limited previous research,^2^^,^^6^^,^^25^ we found that schools accounted for only 1.4%–2.4% of the variability in mental health of early adolescents. Several factors explained this between-school variability; most related to the broader school context and characteristics of the pupil population, rather than operational features of the school. Specifically, schools in urban locations, with a greater proportion of adolescents eligible for free school meals and with more White British pupils, were attended by pupils with poorer mental health.

Urban living is associated with greater income inequality, familial isolation, and exposure to substance abuse, violence, and crime as well as lower community cohesion, all of which are related to the higher prevalence of mental health problems often detected in urban populations.^26^ There is similarly a long-established relationship between socioeconomic adversity and poor childhood mental health.^4^^,^^5^^,^^27^ The mechanisms by which deprivation influences mental health in childhood are multifaceted and incompletely understood, but likely involve parental mental health, family function, nutrition, and sleep, among others.^27^ The increase in mental health inequalities seen in the 21st century in higher-income countries, particularly in relation to emotional problems, is likely to be exacerbated by the disproportional impact of COVID-19 on youths and families and facing debt and financial strain.^28^^,^^29^ Furthermore, socioeconomic and health inequalities may be even wider in urban areas^26^ and are anticipated to increase as a result of the COVID-19 pandemic.^27^, ^28^, ^29^ A public mental health approach that encompasses community as well as school mental health is essential to prevent further deterioration in the mental health of children and adolescents.

The finding that children attending schools with a higher proportion of White pupils had poorer mental health than children in schools with more ethnically diverse pupil populations is surprising. Earlier studies from the United Kingdom suggest that young people from ethnic minorities had a higher prevalence of mental health conditions,^4^ but the results of the present study echo recent large mental health surveys of children and adolescents in the United Kingdom.^5^^,^^29^ Recent austerity policies in the United Kingdom have resulted in drastic reductions in support for children, families, and schools, which were previously less accessed or accessible to ethnic minorities.^30^ Young people from ethnic minorities may therefore have been less adversely affected by these policies. In addition, there is some evidence that psychological distress may be related to ethnic density. Specifically, there could be a possible beneficial effect of more culturally diverse environments for minority students, but majority students seem to be insensitive to this effect.^31^^,^^32^ Finally, the meaning of ethnicity varies greatly with culture, time, and geography, and our findings raise interesting questions about the roles of ethnic diversity and ethnic minority status as influences on mental health of pupils, which require further empirical study.

The only operational, and thus obviously tractable, feature of schools associated with mental health of young people was teacher-rated school climate. Researchers are increasingly encouraged to define school climate as a construct that encompasses school engagement, safety, and environment, both physical and social.^33^ School climate predicts key educational outcomes^7^ as well as mental health^7^ and well-being^12^ of both staff and pupils.^34^ A recent systematic review of school climate interventions concluded that interventions aiming to promote social-emotional learning and school-wide positive behavior programs seemed more effective than those focusing on bullying, community development, or teachers’ working conditions.^34^ However, few of the 18 experimental studies detected were sufficiently methodologically rigorous, and the outcome of primary interest was perception of teachers and pupils of school climate. Another systematic review concluded that there was a clear association between school climate and pupils’ mental health, but as most of the 48 studies were observational and cross-sectional, we cannot claim a causal relationship.^35^ The authors also suggest that future research should pay greater attention to the components that comprise both constructs, well-being and poor mental health, and school connectedness, safety, academic environment, and peer relationships and examine how these interact.

As suggested, theory-driven studies are needed that follow children over several years to examine how broader school context (eg, deprivation), school characteristics (eg, ethnic composition), school operational features (eg, school climate), and pupils’ individual factors (eg, psychopathology) interact to shape the trajectory of mental health of young people over time (Figure S1, available online).^35^ Such frameworks could also be used to examine how SEL and targeted interventions may be more or less effective in certain contexts, schools, and subpopulations of pupils. In this sense, studies should ideally be designed to enable inferences about causality that can shape both policy and intervention development.

While the direct influence of schools on mental health seems to be small, this does not negate schools as a setting in which mental health can be improved via universal and targeted interventions. Furthermore, these small school-level effects may translate into more significant impacts if the substantial future health, economic, and societal costs of poor mental health in adolescence were modeled.^4^^,^^6^^,^^36^ Indeed, there is a growing evidence base that school-level interventions can enhance resilience and functioning of young people, and for young people living in deprived areas, such interventions may be particularly important.^1^^,^^34^ Prospective interventional research is needed to explore how broader contextual and school variables interact with interventions to effect changes in mental health of young people during key developmental windows.^5^, ^6^, ^7^, ^8^, ^9^^,^^12^, ^13^, ^14^, ^15^, ^16^, ^17^^,^^35^^,^^37^ This is something we are doing in our larger MYRIAD study,^17^ which is collecting data from these schools over 2 years so that we will be able to examine the associations over time between the broader school context, school characteristics and operational features, and mental health and well-being of young people.

Regarding study limitations, we recognize that our sample excluded schools that inspections had classified as inadequate or that had no SEL strategy. The inclusion of these poorly functioning schools might have increased the proportion of variation in pupils’ mental health attributable to the school level. Schools were representative of schools across the United Kingdom, but these were schools that had demonstrably good Personal, Social, Health, and Economic Education and participated in a trial. We included private schools, but in the United Kingdom, these institutions serve only 5%–7% of the population, an insufficient number to support a subgroup analysis. Future studies should oversample from uncommon types of schools to study if different types of provision may differ in their influence on mental health.

The usual caveats of how populations vary across a country apply to generalizing outside the United Kingdom. However, our findings are consistent with the reported proportion of variation at the school level in other similar studies, including some in other countries.^13^^,^^14^^,^^3^,^1^^,^^37^^,^^38^ School-level influences on pupil mental health may be observable only in pupils with significant problems, although this was not supported by our sensitivity analysis. Our sample cannot represent pupils who were excluded before commencement of the study by their parents or by their school. Furthermore, we lacked data on some potentially important variables, such as family socioeconomic status, academic attainment, school-level violence, and pubertal status, all of which might influence mental health and well-being. Finally, our measure of school climate was based on teacher ratings alone, while a measure that included pupil, parent, and teacher ratings might have added different and valuable perspectives.^34^

Our findings converge with others to suggest that in early adolescents 11–14 years of age, school influences explain 1.4%–2.4% of the variance in mental health and well-being. These small school-level effects may reflect a relative uniformity across schools in the United Kingdom in current approaches to pupil mental health. In schools located in urban areas, with pupils from predominantly White, disadvantaged backgrounds, poorer mental health in early adolescence is observed. At a population level, such findings are potentially important. Policy and system interventions focused on deprivation are likely to yield improvements in mental health of young people. In terms of schools, our findings converge with others to suggest the importance of school climate to support mental health and well-being in young people. In summary, this study has examined school structural and social features, both of which have important implications for guiding policy and the targeting of interventions.
