# Supplementary material for: The Role of Schools in Early Adolescents’ Mental Health: Findings From the MYRIAD Study
Source: J Am Acad Child Adolesc Psychiatry. 2021 Dec;60(12):1467–78. doi: 10.1016/j.jaac.2021.02.016 (PMC8669152; doi:10.1016/j.jaac.2021.02.016)
Supplement: Figure S1 [file mmc1.docx]

**Figure S1: Schematic Diagram of the Three Types of School-Related Factors.**


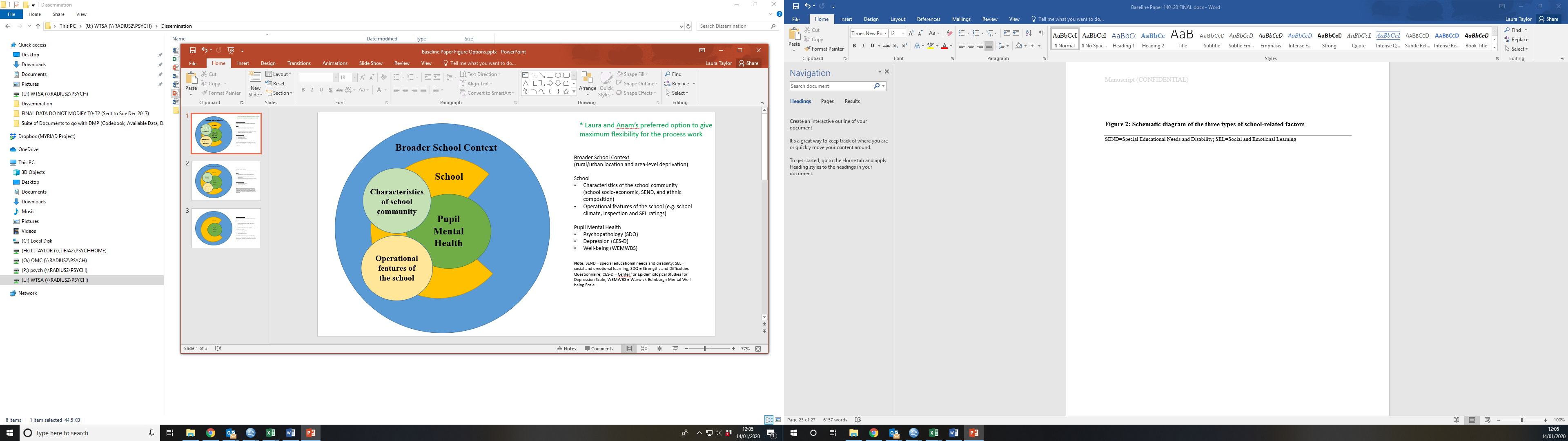


**Note.** Broader School Context: rural/urban location and area-level deprivation. School: Characteristics of the school community (school socio-economic, SEND, and ethnic compositions); Operational features of the school (e.g. school climate, inspection and SEL ratings). Pupil Mental Health: Psychopathology (SDQ); Depression (CES-D); Well-being (WEMWBS). CES-D = Center for Epidemiologic Studies Depression Scale; SEL = social and emotional learning; SEND = special educational needs and disability; SDQ = Strengths and Difficulties Questionnaire; WEMWBS = Warwick-Edinburgh Mental Well-being Scale.
