# Supplementary material for: The Role of Schools in Early Adolescents’ Mental Health: Findings From the MYRIAD Study
Source: J Am Acad Child Adolesc Psychiatry. 2021 Dec;60(12):1467–78. doi: 10.1016/j.jaac.2021.02.016 (PMC8669152; doi:10.1016/j.jaac.2021.02.016)
Supplement: Supplement 1 [file mmc2.docx]

**Supplement 1: Additional Details of Study Methods**

**Study Design**

Collection of baseline data occurred before randomisation and before pupils or teachers were exposed to any of the trial interventions. The trial from which this data is drawn was registered with the ISRCTN in 2016 (ref: 86619085) and will be complete in 2021. Further information can be found in the trial protocol.^1^

**Recruitment and Procedure**

We initially contacted all eligible UK secondary schools via email and letter, as well as a variety of recruitment techniques, such as promoting the research at educational conferences. We targeted later recruitment efforts at schools with characteristics that were under-represented in our sample (e.g., Independent schools and schools in Scotland and Wales), to ensure generalisability. Recruitment of schools was broadly representative of schools across the UK (see Table 1 for school characteristics and ^2-8^ for sources of national demographic data).

Headteachers consented to their school’s participation and a minimum of five qualified or experienced teachers additionally consented to participate and complete study measures. Schools were eligible to participate if they were a mainstream school (not special schools or alternative settings), had not been rated by national inspectors on measures of quality as ‘inadequate’, had a substantive head, could deliver the intervention if randomised to that arm of the trial, had a strategy and structure in place for delivery of SEL (PSHE in England), and had an English speaking curriculum. Schools provided study information to the parents or guardians of pupils in relevant year groups, who then provided opt-out consent for their child’s completion of study measures. At the time of recruitment, pupils were in year 7 (ages 10‒12) and 8 (ages 12‒14) in English schools, and their equivalent years in Northern Ireland, Scotland, and Wales. Year 7 is the first year of secondary school in England, and year 8 is the second. In our sensitivity analyses, pupils aged 12‒14 in Scotland (year 8 in England) were included with the younger year group (year 7 in England, ages 10‒12) because in Scotland this is their first year of high school. We sought assent from all pupils with parental consent (where parents had not opted their child out of the study) at the first data collection visit (baseline). There were no formal exclusion criteria for pupils, other than an inability to provide informed assent and be able to understand English, or, in rare cases, school judgement that the pupil was otherwise unable to take part. Very few pupils were excluded on this basis (see Figure 1).

We collected pupil data in researcher-supervised data collection sessions under exam conditions. Pupils entered their responses directly onto an online system linked to the study clinical trials unit (or on paper in the case of technical difficulties, which were then double entered into the same online system). Where possible, researchers revisited schools to provide a second opportunity to gather data from absent pupils. Teachers completed their corresponding measures individually through a secure online system following provision of informed consent.

Schools, teachers, and pupils were not remunerated for their participation. As part of the wider trial, teachers were remunerated for providing assessments of their pupils’ well-being and executive function (this data is post-randomisation and was not used for the current study).

1. Kuyken W, Nuthall E, Byford S, et al. The effectiveness and cost-effectiveness of a mindfulness training programme in schools compared with normal school provision (MYRIAD): study protocol for a randomised controlled trial. *Trials* 2017; **18**(1): 194.
2. Bibby, P., Brindley, P. (2013) ‘Urban and Rural Area Definitions for Policy Purposes in England and Wales: Methodology (v1.0)’. Available at: https://assets.publishing.service.gov.uk/government/uploads/system/uploads/attachment_data/file/239477/RUC11methodologypaperaug_28_Aug.pdf Last Accessed: 29th July 2019
3. Department for Communities and Local Government (2015) ‘The English Index of Multiple Deprivation (IMD) 2015 – Guidance’. Available at: https://assets.publishing.service.gov.uk/government/uploads/system/uploads/attachment_data/file/464430/English_Index_of_Multiple_Deprivation_2015_-_Guidance.pdf Last Accessed: 29th July 2019
4. Northern Ireland Statistics and Research Agency (2016). ‘Technical Guidance on production of official statistics for Settlements and Urban-Rural Classification’. Available at: https://www.nisra.gov.uk/sites/nisra.gov.uk/files/publications/settlement15-guidance.pdf Last Accessed: 29th July 2019
5. Northern Ireland Statistics and Research Agency (2017) ‘Northern Ireland Multiple Deprivation Measures 2017’. Available at: https://www.nisra.gov.uk/sites/nisra.gov.uk/files/publications/NIMDM17-%20with%20ns.pdf. Last Accessed: 29th July 2019
6. Rural and Environment Science and Analytical Services Division Scottish Government (2016) ‘Scottish Government Urban Rural Classification’. Available at: https://www.gov.scot/publications/scottish-government-urban-rural-classification-2016/ . Last Accessed: 29th July 2019
7. Scottish Government (2016) ‘Introducing: The Scottish Index of Multiple Deprivation 2016’. Available at: https://www2.gov.scot/Resource/0050/00504809.pdf. Last Accessed: 29th July 2019
8. Statistics for Wales (2014) ‘Welsh Index of Multiple Deprivation (WIMD) 2014 Revised’. Available at: https://gweddill.gov.wales/docs/statistics/2015/150812-wimd-2014-revised-en.pdf. Last Accessed: 29th July 2019
