# Supplementary material for: The Role of Schools in Early Adolescents’ Mental Health: Findings From the MYRIAD Study
Source: J Am Acad Child Adolesc Psychiatry. 2021 Dec;60(12):1467–78. doi: 10.1016/j.jaac.2021.02.016 (PMC8669152; doi:10.1016/j.jaac.2021.02.016)
Supplement: Supplement 2 [file mmc3.docx]

**Supplement 2: Additional Detail of School-Level Measures**

**Characteristics of School Population**

All *broader school context* measures were obtained using publicly available data published by the constituent nation the participating schools resided within. Data was usually obtained online from the education and statistics departments (e.g., Department of Education, England). In all cases, published publicly available data were collected according to its proximity to the year in which participating pupils provided questionnaire data.

Government defined urban/rural classification was used to define the urbanity or rurality of the areas the participating schools reside within. Each constituent nation uses their own criteria and parameters most relevant to their nation in order to define urban versus rural areas. Dependant on the nation, this information is available in varying degrees of specificity. In order to draw direct comparison between participating schools, government defined dichotomous categorisation was obtained and used for analysis.

Deprivation level of the participating schools was measured using the Multiple Index of Deprivation 2015 (IMD), which is the official measure of relative deprivation in the UK. Deprivation is cumulative and multi-dimensional and IMD applies a single score (0-10) to all locations in each of the nations in the UK. School postcodes were used to obtain IMD scores to define the context of deprivation the school resides within. Each of the four constituent nations use distinct and varied definitions of deprivation designed to accommodate the targeting of resources. For this reason, IMD scores were obtained directly from the governing body the school belongs to without mapping the nations onto each other.

Characteristics of the *school community* and *operational features of the school* were all obtained using data collected and released by the government as part of the annual school census. All public schools in the UK are required to provide data to their local authority yearly, which is then processed by the relevant nations governing education or statistical department.

**School Quality Ratings**

Each country within the United Kingdom has its’ own schools inspectorate, each with a different quality rating system. To allow for analysis of school quality ratings across schools we mapped them all onto a single quality rating system. The majority of the schools in the project are inspected by Ofsted (England’s state-funded schools inspectorate) with only 12 schools evaluated by a different inspectorate. It was therefore decided the 12 schools would have their ratings mapped onto the Ofsted rating system^1^. Two researchers independently analysed the 12 schools’ inspectorate reports and assigned them a quality rating from 1–4 to be in line with Ofsted quality ratings (no schools met the criteria for the poorest quality rating, ‘Inadequate’). The most recent inspection report for each school, prior to stratification, was used. The Ofsted School Inspection Handbook 2018 informed the researchers’ mappings. The inter-rater reliability for mappings was 91%.

**Assessment of Quality of SEL (or Equivalent) in Study Schools**

The provision of SEL in England is taught in ‘Personal, Social, Health and Economic Education’ (PSHE) lessons. As PSHE is a non-statutory subject in England, there is a high level of variation between schools regarding teaching time allocated to delivering PSHE and the content covered. A researcher gathered data on the provision of PSHE or equivalent in the baseline academic year. These data were obtained from a key informant, usually the staff member with responsibility for this curriculum area, or a member of the school senior leadership team. We assessed provision against 16 quality indicators, created for this trial and identified through review of existing measures and via expert consultation. We assigned schools a score (out of 16) reflecting the number of quality indicators present, with subscale scores reflecting quality in the domains of *Leadership and Strategic Approaches to PSHE*, *Curriculum Content and Delivery* and *Assessment*, *Evaluation, and Consultation*^2^.

**School Climate**

Participating teachers completed three subscales from the Alaska School Climate and Connectedness Survey^3^, through an online data collection system at baseline. These subscales assessed *School leadership and Involvement* (8 items, rated between 1 and 5, Cronbach’s alpha, *α*=0.93), *Staff Attitudes* (5 items rated between 1 and 5, *α*=0.86) and *Respectful Climate* (5 items, rated between 1 and 5, *α*=0.86), with higher scores reflecting a more positive school climate. Although school climate data were obtained from individual teachers, we averaged data across informants within each school to create a composite rating of school climate. We did not have enough teacher-student data for clustering of students by teacher so we used the mean of teacher-ratings at each school as a school-level measure of school climate.

1. Ofsted. Handbook for inspecting schools in England under section 5 of the Education Act 2005. 2018. https://assets.publishing.service.gov.uk/government/uploads/system/uploads/attachment_data/file/730127/School_inspection_handbook_section_5_270718.pdf (accessed 22nd March c2019).
2. Kuyken W, Crane C, Aukland L, Sonley A, Lord L, Weare C. School PSHE Quality Assessment Tool. In Preparation (further details of the measure are available from the authors on request).
3. Association of Alaska School Boards. School climate and connectedness survey [measurement instrument]. In: AASB, editor. Alaska: Juneau; 2015.
