# Supplementary material for: The Role of Schools in Early Adolescents’ Mental Health: Findings From the MYRIAD Study
Source: J Am Acad Child Adolesc Psychiatry. 2021 Dec;60(12):1467–78. doi: 10.1016/j.jaac.2021.02.016 (PMC8669152; doi:10.1016/j.jaac.2021.02.016)
Supplement: Tables S1-S6 [file mmc4.docx]

**Table S1: Number of Schools with Missing Data for each School-Level Variable**

| **Variable** | **n** | **%** | **N** |
| --- | --- | --- | --- |
| Urbanicity | 0 | 0 | 85 |
| IMD | 0 | 0 | 85 |
| Percentage of pupils eligible for free school meals | 0 | 0 | 85 |
| SEND support | 6 | 7.1 | 79 |
| White British percent | 9 | 10·6 | 76 |
| School Sex | 0 | 0 | 85 |
| Number of pupils | 0 | 0 | 85 |
| Pupil-teacher ratio | 9 | 10.6 | 76 |
| School quality, OFSTED ^a^ | 4 | 4.7 | 81 |
| SEL provision | 1 | 1.2 | 84 |
| Teacher-rated school climate, SCCS | 4 | 4.7 | 81 |

**Note**. CESD = Center for Epidemiologic Studies Depression Scale; IMD = index of multiple deprivation; SCCS = School Climate and Connectedness Survey; SEL = social and emotional learning; SEND = special educational needs and disability; OFSTED: Office for Standards in Education; WEMWBS = Warwick-Edinburgh Mental Well-being Scale; SDQ = Strengths and Difficulties Questionnaire.

^a^ OFSTED operates in England only.

**Table S2: Number of Participants with Missing Data for each Individual Variable**

| **Variable** | **n** | **%** | **N** |
| --- | --- | --- | --- |
| Age | 0 | 0 | 26886 |
| Gender | 642 | 2.4 | 26244 |
| Ethnicity | 745 | 2.8 | 26141 |
| Year group | 11 | 0 | 26875 |
| Language | 668 | 2.5 | 26218 |
| WEMWBS | 422 | 1.6 | 26464 |
| CESD | 336 | 1.2 | 26550 |
| SDQ | 582 | 2.2 | 26304 |

**Note**. CESD = Center for Epidemiologic Studies Depression Scale; IMD = index of multiple deprivation; SCCS = School Climate and Connectedness Survey; SEL = social and emotional learning; SEND = special educational needs and disability; OFSTED: Office for Standards in Education; WEMWBS = Warwick-Edinburgh Mental Well-being Scale; SDQ = Strengths and Difficulties Questionnaire.

^a^ OFSTED operates in England only.

**Table S3: Sensitivity Analysis Comparing School-Level Variance in pupil mental health in Year 7 and Year 8**

| **Pupil’s mental health** | **Year 7** | | | | **Year 8** | | | |
| --- | --- | --- | --- | --- | --- | --- | --- | --- |
|  | **Pupils (n)** | **Schools (n)** | **ICC**  **(95% CIs)** | **p value** | **Pupils (n)** | **Schools (n)** | **ICC**  **(95% CIs)** | **p value** |
| Psychopathology (SDQ) | 13396 | 84 | 0.032  (0.024 - 0.040) | < 0.0001 | 12896 | 81 | 0.018  (0.012 - 0.024) | < 0.0001 |
| Depression (CES-D) | 13586 | 84 | 0.019  (0.013 - 0.026) | < 0.0001 | 12952 | 81 | 0.014  (0.009 - 0.020) | < 0.0001 |
| Well-being (WEMWBS) | 13526 | 84 | 0.017  (0.011 - 0.023) | < 0.0001 | 12926 | 81 | 0.014  (0.008 - 0.020) | < 0.0001 |

**Note.** Pupils in Scottish schools in year ‘S1’ were coded as equivalent to English year 7 pupils (although their age maps onto English year 8 pupils) as S1 is their first year in high school. Multilevel models are based on complete case analysis; total sample (schools = 85; pupils = 26,885) but n varies due to missing data. CES-D = Center for Epidemiologic Studies Depression Scale; ICCs: intra-class correlation coefficients (using unadjusted models). SDQ = Strengths and Difficulties Questionnaire; WEMWBS = Warwick-Edinburgh Mental Well-being Scale; 95% CI: 95% confidence interval.

**Table S4: Sensitivity Analysis Comparing School-Level Variance in Pupil Depression and Wellbeing in Psychopathology (SDQ) Non-Cases and Cases**

| **Pupil’s mental health** | **Non-cases** | | | | **Cases** | | | |
| --- | --- | --- | --- | --- | --- | --- | --- | --- |
|  | **Pupils (n)** | **Schools (n)** | **ICC**  **(95% CIs)** | **p value** | **Pupils (n)** | **Schools (n)** | **ICC**  **(95% CIs)** | **p value** |
| Depression (CES-D) | 21042 | 85 | 0.009  (0.006 - 0.013) | < 0.0001 | 5195 | 85 | 0.015  (0.002 - 0.029) | < 0.0001 |
| Well-being (WEMWBS) | 21040 | 85 | 0.010  (0.006 - 0.014) | < 0.0001 | 5195 | 85 | 0.014  (0.00 - 0.028) | < 0.0001 |

**Note.** Non-cases were defined as scoring normal or borderline, whereas cases were defined as scoring high or very high on the SDQ. ICCs are not reported for the outcome of psychopathology as SDQ scores were used to define the non-cases and cases. Multilevel models are based on complete case analysis; total sample (schools = 85; pupils = 26,885) but n varies due to missing data. CES-D = Center for Epidemiologic Studies Depression Scale; ICCs: intra-class correlation coefficients; WEMWBS = Warwick-Edinburgh Mental Well-being Scale. ICCs using unadjusted models; 95% CI: 95% confidence interval.

**Table S5: Intra-Class Correlations for School-Level Variance of Pupils’ Mental Health for England only**

| **Pupil’s mental health** | **N** | | **Unadjusted models** | | **N** | | **Adjusted models for pupil’s age, gender, and ethnicity** | |
| --- | --- | --- | --- | --- | --- | --- | --- | --- |
|  | **Pupils** | **Schools** | **ICC**  **(95% CIs)** | **p value** | **Pupils** | **Schools** | **ICC**  **(95% CIs)** | **p value** |
| Psychopathology (SDQ) | 24286 | 75 | 0.023  (0.018 - 0.027) | < 0.0001 | 24117 | 75 | 0.019  (0.015 - 0.024) | < 0.0001 |
| Depression (CES-D) | 24517 | 75 | 0.015  (0.011 - 0.019) | < 0.0001 | 24068 | 75 | 0.013  (0.009 - 0.017) | < 0.0001 |
| Well-being (WEMWBS) | 24438 | 75 | 0.012  (0.008 - 0.016) | < 0.0001 | 24063 | 75 | 0.011  (0.008 - 0.014) | < 0.0001 |

**Note.** Multilevel models are based on complete case analysis; total sample (schools = 75; pupils = 24,842) but N varies due to missing data. CES-D = Center for Epidemiologic Studies Depression Scale; ICCs: intra-class correlation coefficients; SDQ = Strengths and Difficulties Questionnaire; WEMWBS = Warwick-Edinburgh Mental Well-being Scale; 95% CI: 95% confidence interval.

**Table S6: Results from Multilevel Models With Random Intercepts Showing Grouped Associations between Different Types of School Factors and Pupils’ Mental Health for England Only**

| **School factors** | | **Psychopathology (SDQ)** | | | | **Depression (CES-D)** | | | | **Well-being (WEMWBS)** | | | |
| --- | --- | --- | --- | --- | --- | --- | --- | --- | --- | --- | --- | --- | --- |
|  |  | **Unadjusted models** | | **Adjusted models for pupil’s age, gender, and ethnicity** | | **Unadjusted models** | | **Adjusted models for pupil’s age, gender, and ethnicity** | | **Unadjusted models** | | **Adjusted models for pupil’s age, gender, and ethnicity** | |
|  |  | **Coefficient (95% CIs)** | ***p*** | **Coefficient (95% CIs)** | ***p*** | **Coefficient (95% CIs)** | ***p*** | **Coefficient (95% CIs)** | **p value** | **Coefficient (95% CIs)** | ***p*** | **Coefficient (95% CIs)** | ***p*** |
| **Broader School Context** | | | | | | | | | | | | | |
|  | Urban vs Rural | 0.39  (-0.33 - 1.12) | 0.29 | 0.50  (-0.16 - 1.16) | 0.14 | 1.04  (0.11 - 1.98) | 0.030 | 0.93  (0.06 - 1.81) | 0.041 | -0.71  (-1.57 - 0.15) | 0.11 | -0.69  (-1.51 - 0.13) | 0.10 |
|  | Area-level deprivation | -0.06  (-0.15 - 0.03) | 0.23 | -0.07  (-0.17 - 0.05) | 0.28 | -0.05  (-0.17 - 0.06) | 0.39 | -0.06  (-0.17 - 0.05) | 0.28 | -0.03  (-0.14 - 0.07) | 0.55 | -0.02  (-0.12 - 0.09) | 0.74 |
| **Characteristics of School Community** | | | | | | | | | | | | | |
|  | Pupils eligible for free school meals (%) | 0.06  (0.03 - 0.09) | <0.001 | 0.06  (0.03 - 0.09) | <0.001 | 0.04  (0.00 - 0.09) | 0.05 | 0.05  (0.01 - 0.09) | 0.011 | -0.03  (-0.06 - 0.01) | 0.17 | -0.04  (-0.07 - 0.00) | 0.041 |
|  | SEND support (%) | -0.01  (-0.06 - 0.04) | 0.70 | 0.00  (-0.05 - 0.04) | 0.89 | -0.04  (-0.11 - 0.03) | 0.26 | -0.03  (-0.09 - 0.03) | 0.36 | 0.01  (-0.05 - 0.07) | 0.63 | 0.01  (-0.05 - 0.06) | 0.86 |
|  | Ethnicity of pupils (%): White | 0.02  (0.01 - 0.03) | <0.001 | 0.01  (0.00 - 0.02) | 0.054 | 0.01  (-0.01 - 0.02) | 0.33 | 0.01  (0.00 - 0.02) | 0.10 | -0.02  (-0.03 - -0.01) | 0.001 | -0.02  (-0.03 - -0.01) | 0.005 |
| **Operational Features of the School** | | | | | | | | | | | | | |
|  | Mixed or single-sex school | 0.03  (-0.80 - 0.85) | 0.95 | 0.08  (-0.68 - 0.85) | 0.83 | 0.82  (-0.26 - 1.90) | 0.14 | -0.14  (-1.20 - 0.92) | 0.80 | -0.17  (-1.21 - 0.87) | 0.75 | 0.45  (-0.53 - 1.43) | 0.37 |
|  | School quality | -0.13  (-0.68 - 0.42) | 0.64 | -0.06  (-0.57 - 0.45) | 0.81 | 0.04  (-0.68 - 0.77) | 0.91 | 0.12  (-0.57 - 0.80) | 0.74 | 0.46  (-0.22 - 1.15) | 0.19 | 0.36  (-0.29 - 1.01) | 0.28 |
|  | School size (per 100 pupils) | -0.07  (-0.15 - 0.01) | 0.11 | -0.07  (-0.15 - 0.01) | 0.10 | -0.13  (-0.25 - -0.02) | 0.025 | -0.13  (-0.24 - -0.01) | 0.024 | 0.04  (-0.08 - 0.16) | 0.49 | 0.03  (-0.06 - 0.13) | 0.52 |
|  | Pupil-teacher ratio | -0.08  (-0.24 - 0.08) | 0.31 | -0.08  (-0.22 - 0.06) | 0.26 | -0.11  (-0.31 - 0.09) | 0.28 | -0.14  (-0.34 - 0.06) | 0.15 | 0.01  (-0.19 - 0.20) | 0.95 | 0.05  (-0.13 - 0.22) | 0.60 |
|  | SEL provision | -0.01  (-0.11 - 0.09) | 0.83 | -0.02  (-0.10 - 0.06) | 0.68 | -0.03  (-0.15 - 0.08) | 0.60 | -0.03  (-0.14 - 0.09) | 0.67 | -0.04  (-0.16 - 0.08) | 0.51 | -0.03  (-0.14 - 0.09) | 0.65 |
|  | Teacher-rated SCCS | -1.12  (-2.22 - -0.02) | 0.05 | -1.20  (-2.22 - -0.18) | 0.024 | -1.25  (-2.70 - 0.20) | 0.093 | -1.27  (-2.64 - 0.10) | 0.076 | 0.51  (-0.86 - 1.88) | 0.47 | 0.59  (-0.71 - 1.88) | 0.37 |

**Note.** Multilevel models are based on available case analysis; total sample (schools = 75; pupils = 24,842) but n varies due to missing data. SCCS = School Climate and Connectedness Survey; CES-D = Center for Epidemiologic Studies Depression Scale; SEL = social and emotional learning; SEND = special educational needs and disability; SDQ = Strengths and Difficulties Questionnaire; WEMWBS = Warwick-Edinburgh Mental Well-being Scale.
