# Supplementary material for: The Role of Schools in Early Adolescents’ Mental Health: Findings From the MYRIAD Study
Source: J Am Acad Child Adolesc Psychiatry. 2021 Dec;60(12):1467–78. doi: 10.1016/j.jaac.2021.02.016 (PMC8669152; doi:10.1016/j.jaac.2021.02.016)
Supplement: Supplemental Material Figure S1 [file mmc5.docx]

**Figure S1: Participant Flow Diagram**

**Note**: ^a^ Numbers represent pupil names who were given to the trial by their schools. However, some pupils had already been excluded/opted-out by the schools prior to this (these numbers are not known). The total number of pupils, according to government statistics, who could have been approached was N=30,389.

## Enrollment

Schools (N=85)

Potential Participants

(N=29,841) ^a^

Excluded (n=340)

♦  Opted out by parents/carer (n=304)

♦  Other reasons (n= 36; e.g., No spoken English)

Schools (N=85)

Eligible for approach (N= 29,501)

Excluded (n=2616)

♦  Absent (n=1079)

♦  Non-Assent (n= 1537)

## Analysis

Schools (N=85)

Pupils providing assent (N= 26,885)
